# A Survey of Validation Strategies for CRISPR-Cas9 Editing

**DOI:** 10.1038/s41598-018-19441-8

**Published:** 2018-01-17

**Authors:** Monica F. Sentmanat, Samuel T. Peters, Colin P. Florian, Jon P. Connelly, Shondra M. Pruett-Miller

**Affiliations:** 1 St. Jude Children’s Research Hospital, Department of Cell & Molecular Biology, Memphis, 38105 USA; 2 Washington University School of Medicine, Department of Genetics, St. Louis, 63110 USA

## Abstract

The T7 endonuclease 1 (T7E1) mismatch detection assay is a widely used method for evaluating the activity of site-specific nucleases, such as the clustered regularly interspaced short palindromic repeats (CRISPR)-Cas9 system. To determine the accuracy and sensitivity of this assay, we compared the editing estimates derived by the T7E1 assay with that of targeted next-generation sequencing (NGS) in pools of edited mammalian cells. Here, we report that estimates of nuclease activity determined by T7E1 most often do not accurately reflect the activity observed in edited cells. Editing efficiencies of CRISPR-Cas9 complexes with similar activity by T7E1 can prove dramatically different by NGS. Additionally, we compared editing efficiencies predicted by the Tracking of Indels by Decomposition (TIDE) assay and the Indel Detection by Amplicon Analysis (IDAA) assay to that observed by targeted NGS for both cellular pools and single-cell derived clones. We show that targeted NGS, TIDE, and IDAA assays predict similar editing efficiencies for pools of cells but that TIDE and IDAA can miscall alleles in edited clones.

## Introduction

Advances in genome engineering have ushered in exciting new opportunities for scientists to understand and enhance biology. Programmable nucleases enable targeted genomic modifications, which can aid in the study of biological mechanisms involved in disease development, drug discovery, personalized medicine, agriculture productivity, and even environmental sustainability^1^. Although several nuclease platforms have been developed, including zinc finger nucleases (ZFNs) and transcription activator-like effector nucleases (TALENs), the recently engineered clustered regularly interspaced short palindromic repeats (CRISPR)-Cas9 system is widely used, despite its more recent implementation. The popularity of the CRISPR-Cas9 platform can be attributed to its conceptually straightforward design: a single cloning step generates a single guide RNA (sgRNA) that directs Cas9-mediated endonuclease activity to the site of interest. This circumvents the multistep cloning process required for the assembly of the protein-based DNA binding domains that direct ZFNs and TALENs. Moreover, unlike Cas9 nucleases, the FokI nuclease domain of ZFNs and TALENs functions as a dimer, requiring the design of two proteins with strict orientation and spacing constraints. In contrast, the only design constraint limiting the commonly used *S. pyogenes* Cas9 nuclease is the requirement of a 5′-NGG-3′ protospacer adjacent motif (PAM) on the target template immediately following the sgRNA target sequence. However, this prerequisite can be made more flexible by using Cas9 orthologs with different PAM sequence specificities^2,3^.

Cas9 binding is PAM-dependent, and upon complementary base pairing of the sgRNA to the DNA target, Cas9 produces a targeted double-strand break in the DNA^4^. This break is then repaired by the endogenous cellular repair machinery and can lead to local insertion and/or deletion events (indels) via the nonhomologous end-joining (NHEJ) pathway or to precise sequence modification via homology-directed repair when a user-defined donor template is provided.

Not all sgRNAs are equally efficacious at directing Cas9-mediated DNA modifications. This is, in part, because some sequence contexts do not permit optimal sgRNA design. Inclusion of preferential bases within the sgRNA sequence (e.g., a guanine at position 20, proximal to the PAM), at the variable position of the PAM site (preference for cytosine), or immediately following the PAM sequence (A/C/T) can positively influence sgRNA activity, but such bases may not be present at the desired target site^5^. Moreover, chromatin structure and particular sequence elements (e.g., high GC-content or low-complexity features) may preclude the targeting of some genomic sequences, affecting sgRNA activity^5–7^. Therefore, validation strategies for quantifying the modification frequencies of CRISPR-Cas9 reagents are typically implemented to evaluate activity.

In this study, we explored the accuracy of four assays (T7E1, TIDE, IDAA and NGS) frequently used to determine the level of activity for a given sgRNA when complexed with Cas9. We compared the rates of editing at 19 loci in human and mouse genomes assayed by targeted NGS and T7E1. We find that because of a low dynamic range and a requirement for DNA heteroduplex formation, the T7E1 assay often incorrectly reports sgRNA activities. We also evaluated the reliability of TIDE and IDAA analysis and found that both methods are very predictive of overall sgRNA activity. However, neither the TIDE nor the IDAA assay predicted both indel size and indel frequency for all edited clones tested. While TIDE accurately predicted all indel sizes from tested clones, TIDE deviated by more than 10% of the targeted NGS predicted indel frequencies in 50% of clones tested. IDAA accurately predicted 25% of both indel sizes and frequencies for the tested clones. Overall, our work defines some of the major limitations and advantages of each of the most commonly used sgRNA validation assays.

## Results

### Targeted NGS improves selection of high activity sgRNAs

The T7 endonuclease 1 (T7E1) is a structure-selective enzyme that detects structural deformities in heteroduplexed DNA^8^. In using this assay to detect CRISPR-Cas9-mediated gene editing, reagents are transfected into cells, and the genomic DNA surrounding the target locus is amplified several days later by PCR. This PCR product is then denatured and recomplexed by heating and subsequent slow cooling. If an aberrant NHEJ event has occurred after CRISPR-Cas9 cleavage, a heteroduplex will form between amplicons of different lengths (e.g., mutant and WT amplicons), leading to a DNA distortion that is recognized and cleaved by T7E1. The banding patterns of the cut products are compared between control and experimental samples to determine the frequency of mutations.

**Figure 1:**
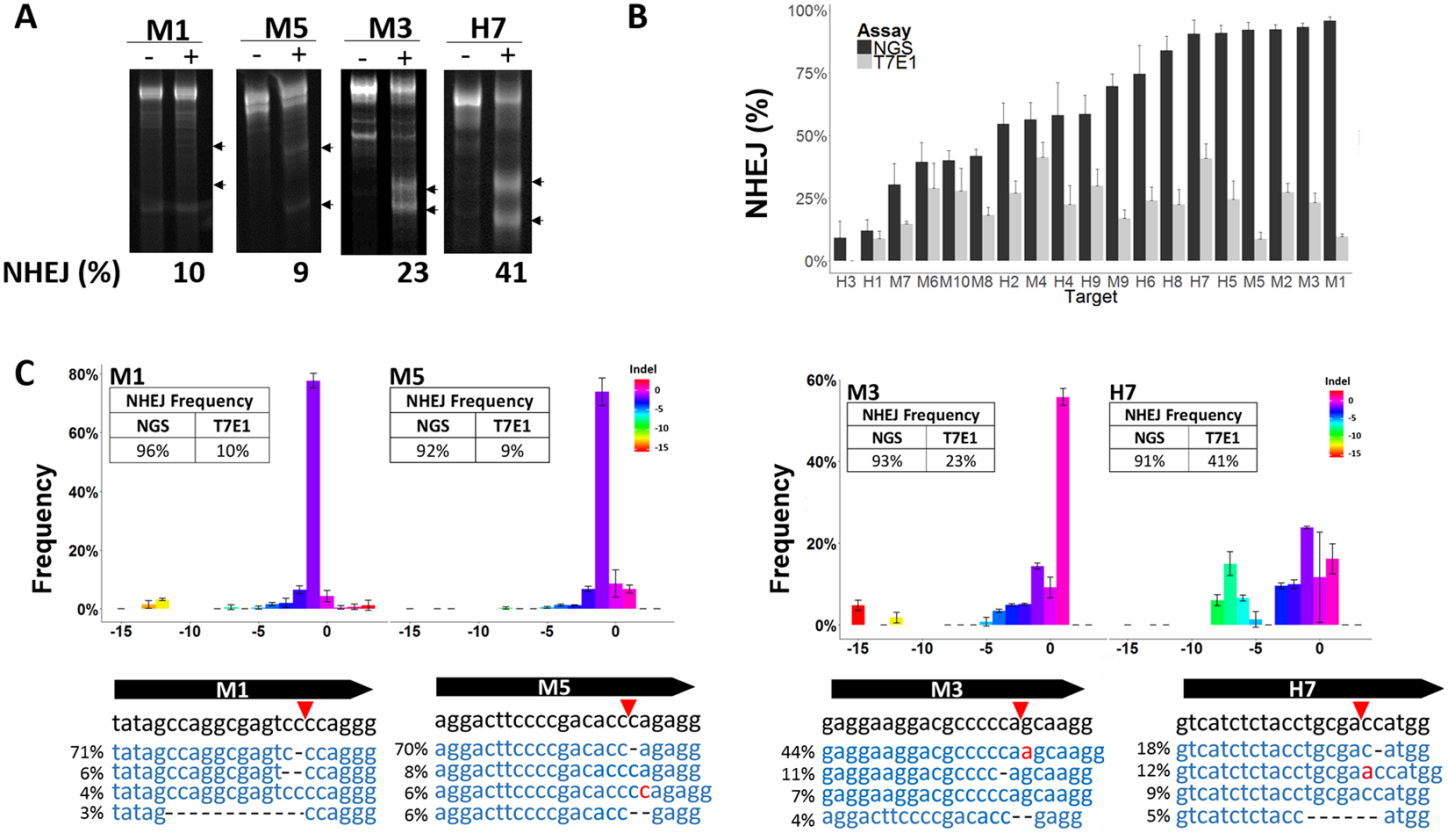
CRISRP-Cas9 activity reported with the T7E1 Assay and Next-Generation Sequencing. (**A**) NHEJ frequency with the T7E1 assay. Representative gel images of T7E1-treated PCR products amplified from the target sites of GFP-negative controls (−) and edited pools (+). (**B**) NHEJ events in CRISPR-Cas9 targets reported by NGS (black bars) or the T7E1 assay (grey bars). Data represent the mean of three biological replicates ±SEM. (**C**) Indel size spectrum (x-axis) and frequency (y-axis) identified by targeted NGS. The top four most prevalent reads are shown below the sgRNA sequence (5′ to 3′) with corresponding deletions (black dashes) and insertions (red letters). Red arrows identify sgRNA cut sites. Data represent the mean of three biological replicates ±SEM.

The T7E1 assay is a cost-effective, technically simple, and easy to interpret method to validate CRISPR reagents. Identified from *Escherichia coli* bacteriophage, T7E1 resolves branched phage DNA for packaging during capsid maturation, and has been shown to cut DNA at the 5′ base of cruciform DNA *in vitro*^9–11^. However, the performance of the assay may be impacted by the length and identity of base pair mismatches, flanking sequence, secondary structure, and relative abundance of mutant sequence^8,12,13^. To determine the accuracy and sensitivity of the T7E1 assay for indels produced by CRISPR-Cas9, we compared the NHEJ frequency estimates derived by the T7E1 assay and targeted next-generation sequencing (NGS). We tested 19 sgRNAs targeting human (H1–H9, Table 1) and mouse (M1–M10, Table 1) genes in K562 and N2a cells, respectively. We used nucleofection to transfect sgRNA and *S. pyogenes* Cas9 (SpCas9) expressing plasmid DNAs and harvested cell lysates 3 to 4 days post-nucleofection. We performed targeted PCR and prepared the resulting amplicons for mismatch detection by T7E1. Although the T7E1 assay is error prone due to subjective bias (e.g., manually choosing troughs to estimate peak area for densitometry) and high background (e.g., excessive banding), predictable banding patterns are apparent in cell pools edited by low- or high-performing sgRNAs. For example, features common to low-activity sgRNAs include parental (i.e., uncut) band intensities that are equal to the negative control and cut products that may be barely visible above background (M1 and M5, Fig. 1A). Alternatively, a feature common to moderate- and high-activity sgRNAs is a reduction in the parental band intensity relative to that of the negative control, concomitant with elevated band intensities in the cut products of expected sizes (M3 and H7, Fig. 1A).

**Table 1:** Summary of sgRNA Targets.

Target	Sequence (5′ → 3′)	GC (%)	Location
H1^a^	TCATATAGTCGCTTTTCTTNGG	35	Coding
H2	AATGGGGACGATTGGGCAAANGG	57	Coding
H3	CTCACCAGTACTCTGCTTTCNGG	50	Noncoding
H4	TGTCTGGGGACACGTCTCCANGG	57	Coding
H5	TCCTCAGCATCTTATCCGAGNGG	52	Coding
H6	GAATGAAAATGCGGTTCTTGNGG	48	Coding
H7	GTCATCTCTACCTGCGACCANGG	57	Coding
H8	GTCCCCTTCTGCCCAATGGTNGG	65	Coding
H9	CCGTCACTGAGACAGTGCGCNGG	65	Coding
M1^b^	TATAGCCAGGCGAGTCCCCANGG	65	Coding
M2	GAGCATAGGCTATGACACAANGG	48	Noncoding
M3	GAGGAAGGACGCCCCCAGCANGG	70	Noncoding
M4	TGGAGACTGTGAAGGTGCTCNGG	57	Coding
M5	AGGACTTCCCCGACACCCAGNGG	35	Coding
M6	ACACTTTATTGTGCTTGTATNGG	35	Splice junction
M7	AACCTCCTCGAACGCGGGAGNGG	70	Coding
M8	TAGCCAAGTGCTACCGCGTANGG	57	Noncoding
M9	CCGGGAATACGACGTGGGCNGG	74	Coding
M10	GGCCTTGGCGTCCTGGTCTTNGG	70	Coding

^a^Human targets are denoted by H1–H9. ^b^Mouse targets are denoted by M1–M10. Sequence, %GC content, and annotated feature (Human GRCh38/hg38 Gencode v24 for human and GRCm38/mm10 GENCODE M14 for mouse).

We observed that the overall frequency of mutations detected by the T7E1 assay for all mouse and human sgRNAs averaged 22%, with 6 of 10 mouse sgRNAs and 5 of 9 human sgRNAs ranging between 17% and 29% (Table S1). The highest activity detected was 41% (Fig. 1B and Table S1). These findings are consistent with those in a previous report, in which a peak T7E1 signal of 37% was achieved with a 50% mix of WT and mutant alleles^12^. We compared our T7E1 results with targeted NGS by creating a tailed library of the PCR amplicons used for the T7E1 assays for 2 × 250 bp sequencing on the MiSeq platform. The NGS data revealed a greater breadth of activity of the sgRNAs and a higher overall NHEJ frequency than did the T7E1 assay. With NGS, an average of 68% sgRNA activity for all mouse and human sgRNAs was detected, and 9 individual sgRNAs yielded indel frequencies of 70% or greater (Fig. 1B and Table S1). Although it was expected that NGS would provide a higher dynamic range, we anticipated the overall trend in activity for all sgRNAs to be consistent between methods. NGS revealed three major sources of inaccuracy in the T7E1 assay. First, poorly performing sgRNAs with less than 10% NHEJ events detected by NGS appeared to be entirely inactive by T7E1 (H3, Fig. 1B). Second, highly active sgRNAs with greater than 90% NHEJ events detected by NGS appeared modestly active in the T7E1 assay (M1 and M5, Fig. 1B). Third, sgRNAs with apparently similar activity detected by the T7E1 assay were actually considerably different when detected by NGS. For example, the M2 and M6 sgRNAs both exhibited ~28% activity in the T7E1 assay, but NGS demonstrated that the M6 pools had half the indel frequency of M2 pools (40% M6 vs 92% M2, Fig. 1B and Table S1). These data are consistent with those in previous reports, suggesting that the T7E1 assay is not reliable when indel frequencies exceed 30%^14^.

**Figure 2:**
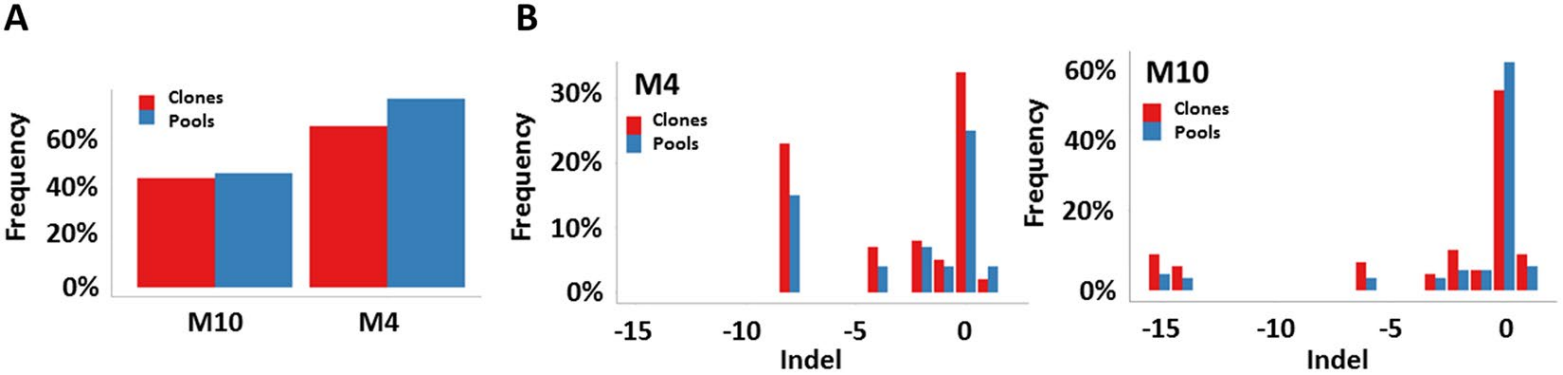
Targeted deep-sequencing of edited pools and single cell derived clones. (**A**) Overall indel frequency measured by targeted deep sequencing for M10 and M4 edited cell pools (blue) and clones (red). (**B**) Comparison of indel sizes and frequencies between M4 and M10 cell pools (blue) and individual clones (red). Indel frequencies for M4 and M10 clones were averaged across 136 and 105 individual clones, respectively.

Targeted deep-sequencing can be used to determine indel frequencies generated by CRISPR-Cas9^15^. However, this PCR-based method is subject to PCR bias for smaller amplicons, template switching, and can result in jackpotting of alleles with large deletions. To determine if NGS of edited pools consisting of 1–15 bp indels accurately reflects true editing efficiency, we compared indel frequencies found in M4 and M10 cell pools to single cell derived clones generated from sequenced pools, 136 and 105 clones, respectively (Fig. 2A). The frequency of indels were comparable between cell pools and clones, as well as across indel sizes – ranging from 1 bp insertions to −15 bp deletions (Fig. 2B). Overall, this data suggests that this NGS approach for assessing editing efficiencies in cell pools can accurately portray individual editing events within pools.

### A lack of indel diversity attenuates T7E1 detection of CRISPR-Cas9 mutations

The T7E1 assay is well suited for detecting indel variants resulting from aberrant NHEJ events, yet single nucleotide polymorphisms are poorly recognized^8,12,16^. To determine how the indel makeup of a pool of edited amplicons generated from cells treated with CRISPR-Cas9 reagents affects T7E1 assay outcomes, we mapped the indel distributions of four highly active sgRNAs with NGS-identified indel frequencies greater than 90%. Two of these sgRNAs exhibited low indel frequencies in the T7E1 assay (M1 and M5), and two exhibited moderate to high activity (M3 and H7) (Fig. 1A). The M1 and M5 sgRNAs displayed representation bias of a −1-bp deletion in more than 70% of reads (Fig. 1C). Both sgRNA sequences contained a dinucleotide homopolymer at the site of the double-strand DNA break (Fig. 1C). This sequence microhomology most likely contributed to the preferential repair outcomes during NHEJ^17^. Such bias in indel composition may be a limiting factor for heteroduplex formation, which is necessary for T7E1 mismatch recognition. Approximately half of the indel population from the M3 sgRNA was composed of a single +1-bp indel. The highest frequency indel from the H7 sgRNA was present in only a quarter of the pool, which yielded a two-fold increase in the T7E1 signal for the H7 sgRNA (Fig. 1B). Although the NGS findings indicated that the M3 and H7 sgRNAs cut equally well (>90% efficiency, Fig. 1C), the T7E1 assay estimated that the H7 sgRNA contained two-fold greater activity than did the M3 sgRNA. Interestingly, the indel size distribution was also wider for the H7 sgRNA, in which a −7-bp deletion accounted for 15% of reads and 3-bp and 2-bp deletions each accounted for 10% of reads. Indel size heterogeneity was greater for the moderately active M3 sgRNA (>20% reads consisted of a 1- to 5-bp deletion, Fig. 1C) than for the poorest performing pools (M1 and M5). These data suggest that pools with a diverse indel population after CRISPR-Cas9 editing more closely reflect the overall editing efficiency as reported by the T7E1 with an upper level of detection at approximately 30–40%.

**Figure 3:**
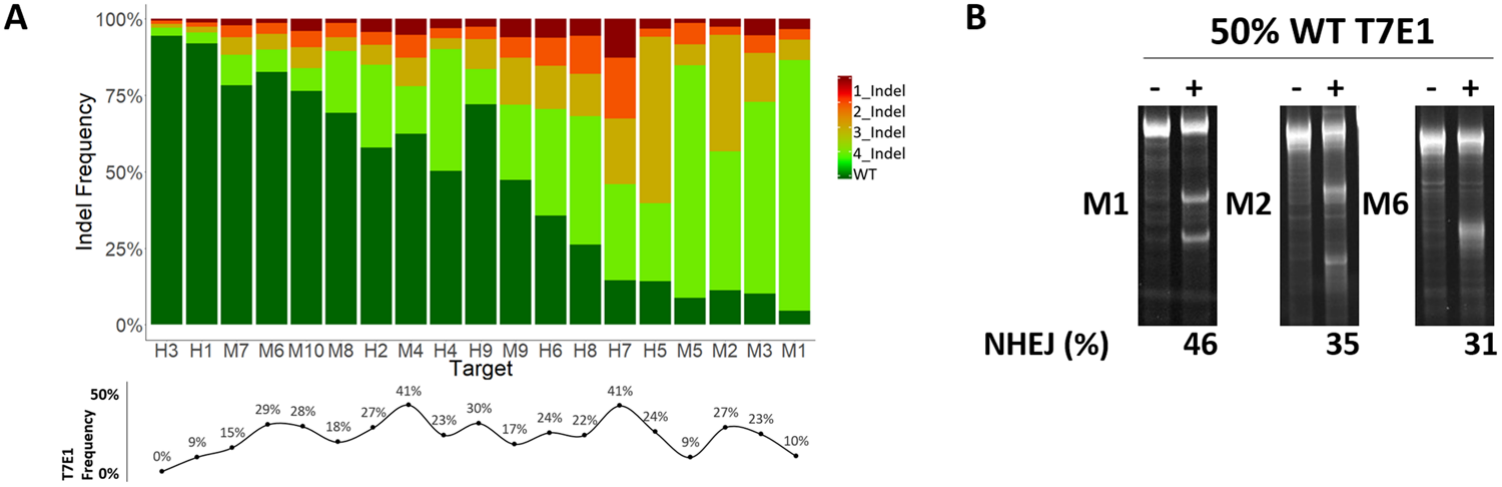
Improved T7E1 sensitivity with amplicon diversity. (**A**) Histogram of the top 4 indels and WT sequence across all targets ordered from lowest to highest in activity reported by targeted deep sequencing. A line diagram of T7E1 frequency data for each target is shown below the histogram for reference (also shown in Fig. 1B). Data represents average of three biological replicates. (**B**) T7E1 assay for M1, M2, and M6 pools heterocomplexed with a 1:1 mixture of amplicons generated from targeted and untargeted wild type (WT) pools. The %NHEJ represents average of two biological replicates.

The T7E1 signal is more associated with indel distribution than with overall sgRNA activity, in which the T7E1 signal was stronger for less indel-biased samples. When indel frequencies are plotted for each sample in ascending %NHEJ order based on NGS data (as in Fig. 1A) two trends emerge that explain the discrepancies between the T7E1 and NGS data (Fig. 3A). First, high activity sgRNAs with a single dominant indel lack sufficient amplicon diversity for an accurate T7E1 assay (e.g. M1 and M5). Second, targets with T7E1 signals above 20% have more widespread distributions across 2 or more indel species (e.g. M4 and H7).

Homoduplexing of high frequency indels most likely attenuates T7E1 sensitivity because the relative availability of WT (or alternate indel) species for heteroduplex formation is scarce. Given that some sgRNAs generated a high proportion (>50%) of single indel species, low T7E1 signals may be a consequence of the lack of heterogenous template in the pooled population.

To test if the limiting determinant for T7E1 sensitivity is the presence of a heterogeneous template, we used a one-to-one ratio of amplicons generated from untargeted and targeted N2a cell pools for heteroduplex formation. We assayed heteroduplexes by T7E1 predicting that pools harboring high NHEJ frequencies (>90% by NGS) with low indel complexities (i.e., M1,a single −1-bp deletion in >70% of amplicons) would most greatly improve. In addition, the abundance of WT amplicons provided by the WT amplicons spike-in would allow for a more accurate quantitative estimate of relative activity across sgRNAs – generating data more congruent with targeted deep-sequencing. Indeed, the one-to-one ratio of WT-to-targeted template resulted in improved detection across all samples tested (Fig. 3B). The M1 pool improved by T7E1 from 10 to 46%, which is the expected T7E1 signal plateau^12^. Pools M2 and M6 also showed T7E1 signals that better reflected their overall NHEJ rates, with M2 at 35% (previously 27%, 92% by NGS) and M6 at 31% (previously 29%, 39% by NGS). Thus, spiking-in WT amplicon prior to heterocomplexing PCR products can increase the accuracy of the T7E1 assay. One caveat of this approach is that low frequency editing may fall below the limit of detection.

### TIDE and IDAA detect indels with similar frequency to targeted NGS in cell pools

**Figure 4:**
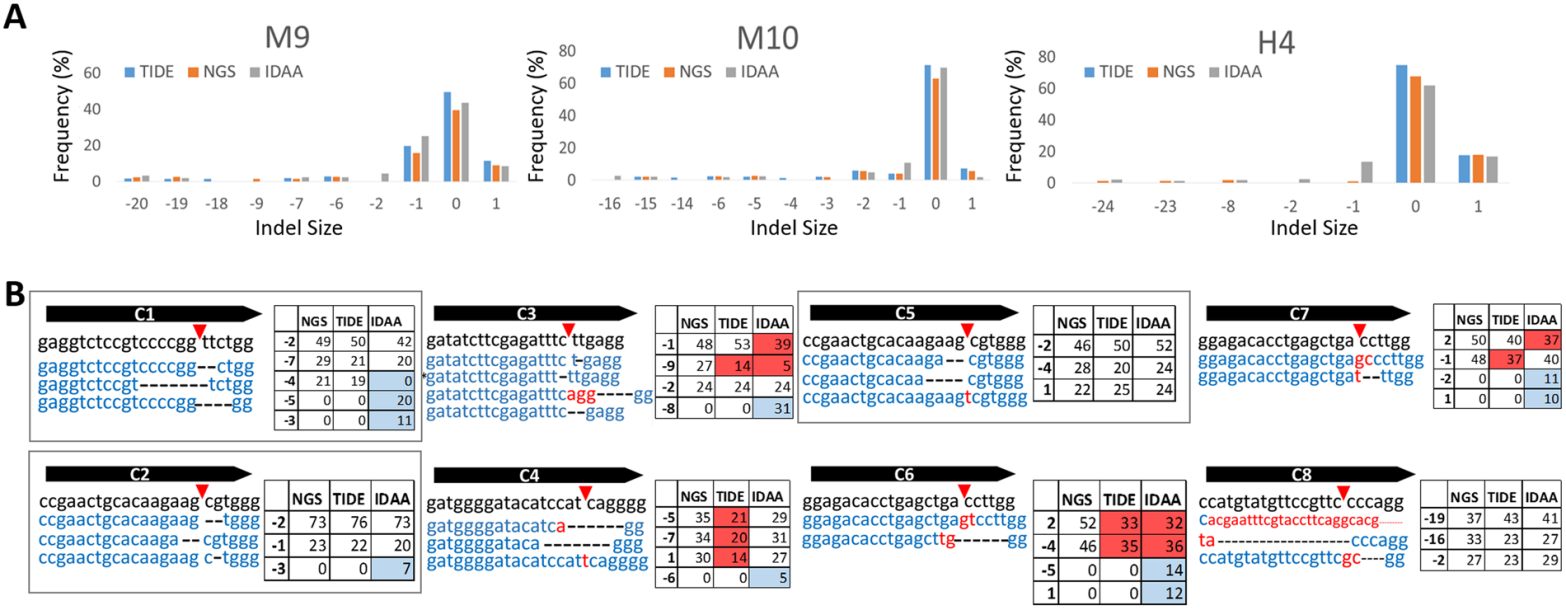
CRISPR-Cas9 activity reported by TIDE-Coupled Sanger Sequencing, Next-Generation Sequencing, and IDAA. (**A**) Indel spectrum and frequency predicted by TIDE, NGS, and IDAA from three pools of edited cells. (**B**) Comparison of NGS, TIDE, and IDAA for eight edited clones. The most prevalent reads (>5%) by targeted NGS are shown below the sgRNA sequence (5′ to 3′) with corresponding deletions (black dashes) and insertions (red letters). Red arrows identify sgRNA cut sites. The allele frequencies determined by NGS, TIDE, and IDAA are depicted in the adjacent table. Allele frequency and indel identity miscalls by TIDE or IDAA are highlighted in red or blue, respectively. Alignments labelled with an asterisk (*) represent indels with insertions and/or deletions that span beyond the sgRNA area represented.

Targeted NGS of edited pools can improve gene-editing efficiency by revealing which sgRNA design yields the greatest activity and desired indel frequency (i.e., out-of-frame indels for gene knockout). Although advances in sequencing technology have greatly reduced cost, NGS remains cost prohibitive for small sample sizes. Recently, the Tracking of Indels by Decomposition (TIDE) algorithm was created to analyze Sanger sequence traces generated from convoluted samples^18^. This method resolves indel size frequencies from edited cell populations by comparing and decomposing Sanger traces made from PCR products of targeted regions from WT and edited templates. We compared NHEJ event estimates and indel frequencies of TIDE-coupled Sanger sequencing and targeted NGS for three sgRNA pools: M9, M10, and H4. All major indels (>5%) identified by NGS were also identified by TIDE at comparable frequencies (Fig. 4A). Although TIDE tended to overestimate the presence of WT amplicon by ~10–20% (Fig. 4A and Supplemental Fig. 1), it was useful in discerning the spectrum and relative frequency of distinct indels after CRISPR-Cas9 editing from pools of edited clones.

A similar and cost-efficient, site-specific nuclease validation assay is the Indel Detection by Amplicon Analysis (IDAA) assay^19^. The IDAA assay uses tri-primer amplicon labeling followed by DNA capillary electrophoresis. In brief, gene-specific primers are used in conjunction with a third FAM-6 labeled primer to amplify and label PCR amplicons obtained from edited genomic DNA. The labeled amplicons are then subjected to capillary electrophoresis, which allows peaks to be called based on size and fluorescence intensity. By comparing labeled amplicons from WT cells to those of edited cells, one can determine the frequency and size of indels present in a population. We compared NHEJ event estimates and indel frequencies of IDAA to that of NGS and TIDE for sgRNA pools M9, M10, and H4. All major indels (>5%) identified by NGS and TIDE were also identified by IDAA at comparable frequencies (Fig. 4A). Similar to TIDE, IDAA tends to overestimate the presence of WT amplicon by ~10–20% (Fig. 4A and Supplemental Fig. 1), but was useful in discerning the spectrum and relative frequency of distinct indels after CRISPR-Cas9 editing from pools of edited clones. One limitation that we observed with the IDAA assay is that we had to manually call the peaks −1-bp and +1-bp from the WT peak as the peak calling software (Peak Scanner 2) was not able to call these peaks. This was due to the peaks being in very close proximity to the WT peak (with fluorescent intensities more than double of any other peak). Moreover, most of the tri-primer amplicons obtained in our experiments (including WT controls) contained a −1 bp “shoulder” indel that is suggested to be due to incomplete 3′ adenine nucleotide addition to the IDAA amplicon^20^ (Supplemental Fig. 2). This results in a higher −1-bp frequency for IDAA amplicons compared to NGS or TIDE (Fig. 4A).

To further compare the sensitivities and dynamic ranges of NGS, TIDE, IDAA, and T7E1 assays, we performed a spike-in experiment in which we added increasing amounts of CRISPR-edited genomic DNA to WT genomic DNA (Supplemental Fig. 1). Overall, in both loci tested, NGS yielded the highest indel frequencies and the highest R^2^ coeffecient (0.9999 and 0.9916). TIDE and IDAA indel frequencies were similar and about 10–20% lower than targeted NGS. The fit of the trend line was less for IDAA largely due to higher than predicted values at samples containing lower amounts of edited genomic DNA. The higher values at the lower indel containing samples were a direct result of the −1 bp “shoulder” observed in other IDAA samples from the incomplete adenine addition (Supplemental Fig. 2). For example, the fit is better for loci 1, which had a minor −1 bp shoulder compared with loci 2. Moreover, a 60 minute final extension time and a proof-reading polymerase were used in efforts to reduce the problematic −1 bp shoulder, but with little improvement^20^. IDAA results may improve with further primer optimization or by using a polymerase without adenine tailing. T7E1 trend lines fit well with an R^2^ coefficient of over 0.96 for both loci. However, T7E1 indel frequencies were similar to the other assays at low amounts of edited genomic DNA, but were significantly less as the amount of edited DNA increased. It should be noted that the indel diversity for both tested loci was high, which likely increased the fit of the trend line for the T7E1 samples (Supplemental Fig. 1B).

### TIDE and IDAA can miscall alleles in edited clones

Identifying edited clones derived from CRISPR-Cas9 treated pools commonly involves TA cloning of PCR products amplified from the edited region, transforming the resultant plasmids into bacterial cells, and picking individual colonies for Sanger sequencing. This process is laborious, time consuming, and not necessarily accurate because cells maintained in culture, particularly cancer cell lines, are often karyotypically abnormal, resulting in unknown copy numbers^21,22^. Therefore, many colonies must be sequenced to obtain full allelic representation. We tested the precision of TIDE-coupled Sanger sequencing and IDAA compared to targeted NGS for the genotyping of a panel of 8 clones produced from various mouse and human cell lines (Fig. 4B). The clones contained distinct indel ratios identified by NGS. Clones with indels containing only a deletion or an insertion per allele were classified as “simple” while clones with concomitant insertions and deletions were classified as “complex”. For simple deletion clones C1, C2, and C5 allelic fractions from NGS suggested the presence of 4 alleles (e.g. C1 2:1:1, C2 3:1, C5 2:1:1). The indel identities and fractions were correctly called by TIDE for all three simple clones, while IDAA miscalled indels for C1 and C2 (Fig. 4B, highlighted in blue). Both TIDE and IDAA miscalled indel sizes or frequencies for all complex clones except clone C8 (Fig. 4B, miscalled indel sizes highlighted in blue, miscalled frequencies highlighted in red). Overall, TIDE was able to correctly call all indel sizes for both simple and complex indels, but TIDE miscalled the indel frequency in 50% of the clones. IDAA correctly called both the indel size and frequency in 25% of clones. The presence of inaccurate frequencies and unexpected indels can confound clone verification and suggest contamination with other clonal populations arising from the same edited pool. Additionally, although both TIDE and IDAA analysis did correctly predict that all 8 edited clones were knockout clones (lacked WT alleles), neither assay specifies the sequence identity of called indels and, consequently, does not eliminate the need for laborious TA cloning and Sanger sequencing procedures to obtain the accurate genotype of a given clone.

## Discussion

These results suggest that the NHEJ frequency estimated by the T7E1 mismatch detection assay is often inconsistent with the actual frequency of editing in pools of cells. The findings of the T7E1 assay were more congruent with those of NGS in heteroduplexed pools containing a lack of indel bias. We found the T7E1 assay had a low and limited detection range that plateaued at 30 to 40% for CRISPR-Cas9 edited pools, which was below the average activity of sgRNAs assayed (63% for all mouse and human guides) and resulted in unreliable NHEJ frequencies across target regions. Dilution of PCR products from targeted regions with WT amplicons increased the accuracy of the T7E1 signal suggesting that high activity sgRNAs that produce a single, dominant indel (e.g. due to local microhomology) may show a low T7E1 signal. Therefore, caution should be applied when using T7E1-based quantitative indel estimates, as comparing various CRISPR-Cas9 design or protocol conditions with the T7E1 assay may lead to inaccurate conclusions. Our results suggest that edited cell pools consisting of high-complexity indels with a diverse range of sizes exert a stronger influence on T7E1 sensitivity than does overall editing efficiency (Fig. 3A).

We also show that indel estimates with TIDE and IDAA are typically congruent with those of targeted NGS, although likely at underestimated frequencies in assayed pools of amplicons. This suggests that TIDE or IDAA can aid in sgRNA selection when particular indels are desired (e.g., out-of-frame or gene-disrupting indels) or when comparing activities of multiple gRNAs. TIDE and IDAA assays was less reliable when performed on individual clones especially those containing complex indels. Additionally, TIDE relies on high quality Sanger sequencing traces, which cannot be easily obtained for every genomic locus. Additionally, TIDE most accurately predicts indels of limited size. For example, the default range for the TIDE algorithm is −10 bp to +10 bp. Going beyond this window reduces the confidence level^18^. TIDE is also limited in sensitivity and can only identify indels up to about 1–2%. With the currently available analysis programs, IDAA requires one to manually look at peaks from a control sample and the edited sample and likely manually call peaks that may overlap in order to get accurate indel representation. Additionally, most of the tri-primer amplicons that we tested yielded a −1 bp shoulder that confounded interpretation of results. Moreover, neither TIDE nor IDAA reports the sequence identity of identified indels, and therefore, does not abrogate the need for additional sequencing in order to determine the genotype of a clone.

In comparing targeted NGS to other activity assays such as the T7E1, TIDE, or IDAA assays, it is important to note that the cost and labor involved in setting up an NGS pipeline, including sequence analysis, may be prohibitive for labs working with few samples and/or sgRNA targets. Although NGS can have high error rates at GC- and AT-rich regions and homopolymer stretches, the overall error rate tends to be low at <0.4%^23^. Indeed, every new NGS platform strives for higher precision at longer read lengths that will one day allow high throughput means to assess larger structural editing events not easily performed with the current technology. It is also important to note that all of the aforementioned assays give useful results in many cases. For example, we have not observed false positive results with the T7E1, TIDE, or IDAA assays. Thus, if a sgRNA is shown to be active by any of these assays, one can have high confidence that there is cutting occurring. All four assays (NGS, T7E1, TIDE and IDAA) are PCR-based assays and therefore have some additional limitations. For example, none of the approaches can detect large mutations such as translocations or large deletions that remove one or both primer binding sites. Additionally, PCR-based assays can show bias for smaller amplicons, and a limited number of amplification cycles should be used when possible.

In conclusion, although the T7E1 assay is commonly used to report genome editing frequencies, we show that this assay often does not accurately portray true sgRNA activities. The TIDE and IDAA assays more closely resemble targeted NGS predicted indel sizes and frequencies for pools of cells and are easily performed by most molecular biology labs. However, both TIDE and IDAA can miscall indel frequencies and/or indel sizes from single-cell derived clones. Additionally, neither assay specifies the sequence identity of identified indels and, consequently, does not eliminate the need for laborious TA cloning and Sanger sequencing procedures to obtain the accurate genotype of a given clone. Moreover, targeted NGS is the optimal tool for assaying edited pools and clones, as it provides accurate estimates of indel size, frequency, and sequence identity.

## Material and Methods

### Cell culture and nucleofection

K562 and N2a cells were obtained from ATCC and cultured in IMDM (Gibco) and MEM (Gibco) basal media, respectively, at 37 °C in 5% CO_2_. Each culture medium was supplemented with 10% FBS (Gibco), GlutaMAX (Gibco), and Penicillin/Streptomycin (Gibco). We transfected 200 ng (0.13 pmol) sgRNA and 500 ng (0.1 pmol) Cas9 pDNA in 2.5 × 10^5^ cells via nucleofection (Lonza 4D-Nucleofector, X Unit). The Cas9 expression vector (p3a-Cas9HC) was a gift from Jin-Soo Kim (Addgene plasmid #43945)^24^. Single cell sorting was performed on the MoFlo Cell Sorter (Beckman Coulter) at the Siteman Flow Cytometry Core at Washington University in St. Louis or at the Flow Cytometry Core at St. Jude Children’s Research Hospital.

### Plasmid construction

All sgRNA plasmids were assembled by annealing complementary oligonucleotides (IDT) of the 20-nucleotide target sequence flanked by *Bsm*BI sites, cutting with *Bsm*BI (NEB), and ligating the product with T7 DNA ligase (Intact Genomics) to the MLM3636 sgRNA expression vector (gift from Keith Joung, Addgene plasmid # 43860), as previously described^25^.

### Mismatch detection assay

Genomic DNA was extracted with 500 µL extraction buffer (10 mM Tris, pH 8; 2 mM EDTA; 0.2% Triton X-100; 200 µg/mL proteinase K) heated at 65 °C for 15 min, followed by 95 °C for 5 min. Target regions were PCR-amplified (see Table S2 for primer sequences) with EconoTaq PLUS GREEN 2X Master Mix (Lucigen) or AccuPrime Taq DNA Polymerase, High Fidelity (ThermoFisher), according to manufacturer protocol. PCR products were denatured at 95 °C for 10 min and re-annealed at −2 °C per second temperature ramp to 85 °C, followed by a −1 °C per second ramp to 25 °C. The heterocomplexed PCR product (5 µL) was incubated with 5 U T7E1 enzyme (New England Bio Labs) at 37 °C for 20 min. Products from mismatch assays were electrophoresed on a Novex 10% TBE gel (Invitrogen). Densitometry analysis was performed with ImageJ^26^. The estimated percent NHEJ was calculated with the following formula:

$$ \% {\rm{NHEJ}}\,{\rm{events}}=100\,\times \,[1-{(1-{\rm{fraction}}\mathrm{cleaved})}^{(1/2)}],$$

where the fraction cleaved is defined as $\frac{(\mathrm{density\; of\; digested\; products})}{(\mathrm{density\; of\; digested\; products}+\mathrm{undigested\; parental\; band})}.$

### Next-generation sequencing

Libraries were made with a two-step PCR protocol, in which the target genomic site of interest was first amplified (Step1) with primer containing partial Ilumina sequencing adaptors followed by a second PCR with primers that contained indices and necessary Illumina sequencing adapters (Step2). Briefly, target regions were amplified with locus-specific primers (Table S2), containing universal 5′ tails on the forward (5′-CACTCTTTCCCTACACGACGCTCTTCCGATCT-3′) and reverse (5′-GTGACTGGAGTTCAGACGTGTGCTCTTCCGATCT-3′) primers. PCR amplifications were performed with EconoTaq PLUS GREEN 2X Master Mix or AccuPrime Taq DNA Polymerase, High Fidelity, according to the manufacturer protocol. Indexing of the Step1 PCR product was performed by using 0.1X volume from Step1 with indexing primers and melting at 94 °C for 2 min, followed by five cycles of 94 °C for 30 sec, 54 °C for 30 sec, and 72 °C for 40 seconds. We generated 2 × 250 reads with the Illumina MiSeq platform at the Center for Genome Sciences and Systems Biology (Washington University) or the Hartwell Center (St. Jude).

### TIDE

Sanger traces were generated by GENEWIZ or the Hartwell Center (St. Jude) with target-specific PCR products and analyzed with the TIDE webtool (http://tide.nki.nl)^18^. Default parameters were used for the analysis with the exception of the indel size range, which was set from 10–35 bp.

### IDAA

The IDAA assay was performed as previously described^19^. In brief, three primers were used to amplify and label each amplicon: a gene specific forward primer with common 5′ tail (5′CACTCTTTCCCTACACGACG 3′), a gene specific reverse primer, and a Fam-6 common forward primer that anneals to the common tail of the gene-specific forward primer (Supplemental Table 2). Primers were used in a 1:10:10 ratio of forward primer: FAM-6 labeled primer: reverse primer. Accuprime HiFi polymerase (ABI/Life Technologies) was used for amplification and touchdown thermocycling conditions were used as previously described^19^. PCRs were diluted 1:10 and mixed with LIZ500 or LIZ600 size standards (ABI/Life Technologies) and applied to fragment analysis on a ABI 3730xl sequencer (ABI/Life Technologies). Data was analysed using Peak Scanner 2 software (ABI/Life Technologies).

### Data Availability

All data generated or analyzed during this study are included in this published article (and its Supplementary Information files.

## Electronic supplementary material

Supplemental Information
