# Supplementary material for: A Survey of Validation Strategies for CRISPR-Cas9 Editing
Source: Sci Rep. 2018 Jan 17;8:888. doi: 10.1038/s41598-018-19441-8 (PMC5772360; doi:10.1038/s41598-018-19441-8)
Supplement: Supplementary file 1 — Supplemental Information [file 41598_2018_19441_MOESM1_ESM.pdf]

SUPPLEMENTAL INFORMATION

A Survey of Validation Strategies for CRISPR-Cas9 Editing

Monica F Sentmanat[2], Samuel T. Peters[1], Colin P. Florian[2], Jon P. Connelly[1], and Shondra M. Pruett-Miller[1,*]

Supplemental Information includes two tables and can be found with this article online.

Target	NGS1	NGS2	NGS3	T7E1_1	T7E1_2	T7E1_3	NGS_Ave	NGS_SEM	T7E1_Ave	T7E1_SEM
M1	0.94	0.94	0.99	0.08	0.12	0.09	0.96	0.02	0.10	0.01
M2	0.92	0.89	0.96	0.32	0.29	0.21	0.92	0.02	0.27	0.03
M3	0.93	0.91	0.96	0.31	0.20	0.19	0.93	0.01	0.23	0.04
M4	0.60	0.43	0.66	0.37	0.53	0.34	0.56	0.07	0.41	0.06
M5	0.88	0.90	0.98	0.13	0.09	0.04	0.92	0.03	0.09	0.03
M6	0.55	0.32	0.31	0.47	0.13	0.27	0.39	0.08	0.29	0.10
M7	0.14	0.35	0.42	0.13	0.15	0.16	0.30	0.08	0.15	0.01
M8	0.41	0.38	0.47	0.15	0.15	0.24	0.42	0.03	0.18	0.03
M9	0.78	0.61	0.70	0.11	0.20	0.20	0.70	0.05	0.17	0.03
M10	0.35	0.62	0.49	0.27	0.13	0.44	0.49	0.08	0.28	0.09
H1	0.18	0.15	0.03	0.05	0.07	0.15	0.12	0.04	0.09	0.03
H2	0.48	0.71	0.45	0.18	0.31	0.33	0.55	0.08	0.27	0.05
H3	0.03	0.02	0.22	0.00	0.00	0.00	0.09	0.07	0.00	0.00
H4	0.32	0.68	0.74	0.08	0.25	0.34	0.58	0.13	0.23	0.08
H5	0.86	0.97	0.90	0.12	0.25	0.37	0.91	0.03	0.24	0.07
H6	0.53	0.93	0.77	0.21	0.34	0.16	0.74	0.12	0.24	0.05
H7	0.80	0.98	0.95	0.34	0.36	0.53	0.91	0.06	0.41	0.06
H8	0.75	0.83	0.94	0.14	0.19	0.34	0.84	0.06	0.22	0.06
H9	0.46	0.59	0.72	0.20	0.27	0.43	0.59	0.07	0.30	0.07

Table S1. Mouse (M1-10) and human sgRNAs (H1-9) with corresponding indel frequencies determined by deep-sequencing (NGS) or T7E1 (T7E1). Three biological replicates (1-3) are represented with their respective averages and SEM.

Target amplification

Primer Name	Sequence (5' -> 3')
M1.DS.F	accccaggagtatctgccccctctca
M1.DS.R	agaagcaggcagagcagagggtggc
M2.DS.F	cttgccaacagctcgacccctgctg
M2.DS.R	tctgggtctgcagacgtggtatcca
M3.DS.F	agcagagagccaacttccacaagtt
M3.DS.R	ggtctgactcagtacaagacaaagcaaagg
M4.DS.F	acccatcatgccctcttgctagccc
M4.DS.R	tgctttggtcatggtgaacagtgact
M5.DS.F	ctagccttgcttagctgcat
M5.DS.R	ctgagggcacttttattgtcctg
M6.DS.F	ggggaaattggtggagtcaatattcttggc
M6.DS.R	cagaccaaaggacggaccaggcagg
M7.DS.F	cttgaaccgtagagaccccaa
M7.DS.R	cagaccaaaggacggaccaggcagg
M8.DS.F	tcattgtcccgtagccttga
M8.DS.R	tgtagtagggcggagcttgg
M9.DS.F	ttccagaggagcggctggggattcg
M9.DS.R	ggggtggggtgtaagcctggagacc
M10.DS.F	agtgcttttcgtgatggacctgacct
M10.DS.R	tggtcgggcagtagggggaattgtc
H1.DS.F	aactgctgctaaaagcaatgtcag
H1.DS.R	ctgaatgaagaaaactgccttcc
H2.DS.F	tctgggccaaagcattttcgtgacca
H2.DS.R	gcctttgcctcttcgatagcgtgct
H3.DS.F	gggcggcgtgggatttc
H3.DS.R	gtccaaatcttcccagaatgcc
H4.DS.F	tggcagggctggtctttctctggca
H4.DS.R	agtcccgagccaaagccgagtgaca
H5.DS.F	catgagcgctgctcagatag
H5.DS.R	cagttgcaaaccagacctca
H6.DS.F	ctggttcagaactttttgtggg
H6.DS.R	ggatccacagctgtcagtttc
H7.DS.F	gtggcatacaacagtagaattggg
H7.DS.R	cagtaccacttacctttggaccta
H8.DS.F	actgtgaactgtgtttccgtt
H8.DS.R	agaccaactcctcccacaaatg
H9.DS.F	gctgcctctgttcttcacct
H9.DS.R	ctgcacagggtgaactcctc
C1.DS.F	gccccggcacaaatcctggctttgt
C1.DS.R	cagcacccccaggaccctgtgatct

C2.DS.F	tctccagcccaccactgtttcagat
C2.DS.R	ccgtctcccaccctatcccctgcct
C3.DS.F	agcatggcctcggtgttccattggt
C3.DS.R	agtcaaagcaatcctcctgcctcaa
C4.DS.F	gttgctacctctaaggcccca
C4.DS.R	cactttctcctacctttgtggca
C5.DS.F	tctccagcccaccactgtttcagat
C5.DS.R	ccgtctcccaccctatcccctgcct
C6.DS.F	ggcgggatgggctttcagaacaccg
C6.DS.R	agggatgcggctcaagggtaagggg
C7.DS.F	ggcgggatgggctttcagaacaccg
C7.DS.R	agggatgcggctcaagggtaagggg
C8.DS.F	tgctcactcctgcaagttaaaaccctggt
C8.DS.R	acagatgaatccccctccacctccca
Common IDAA primer	cactctttccctacacgacg

Table S2. List of primers for PCR amplification of target regions used for T7E1 and NGS

gRNA Sequences

**Clone**	**Sequence (5' -> 3')**
C1	gaggtctccgtccccggttc
C2 and C5	ccgaactgcacaagaagcgt
C3	gatatcttcgagatttcttg
C4	gatggggatacatccatcag
C6 and C7	ggagacacctgagctgacct
C8	ccatgtatgttccgttcccc
IDAA primers	
pool 1	gtactgtgtcagggtactag
pool 2	aaattccaggttgtcatcaa
M9	ccgggaatacgacgtgggcg
M10	ggccttggcgtcctggtctt

Table S3. List of sgRNA sequences used to generate clones shown in Figure 4b and

Supplemental Figure 1.

A

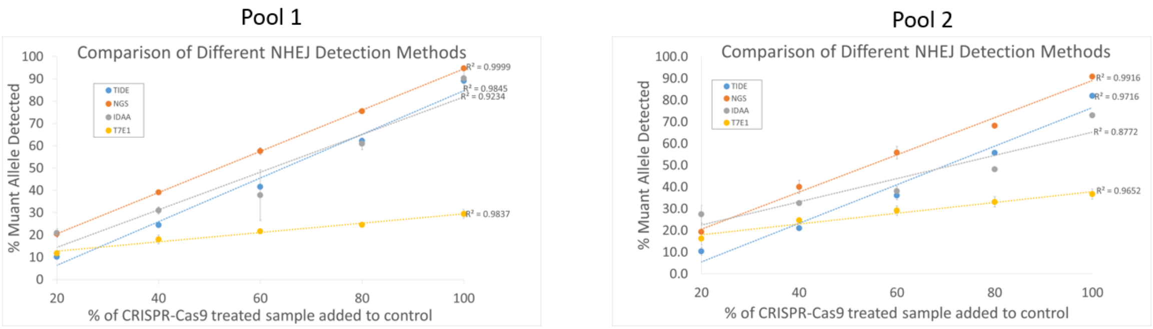

B

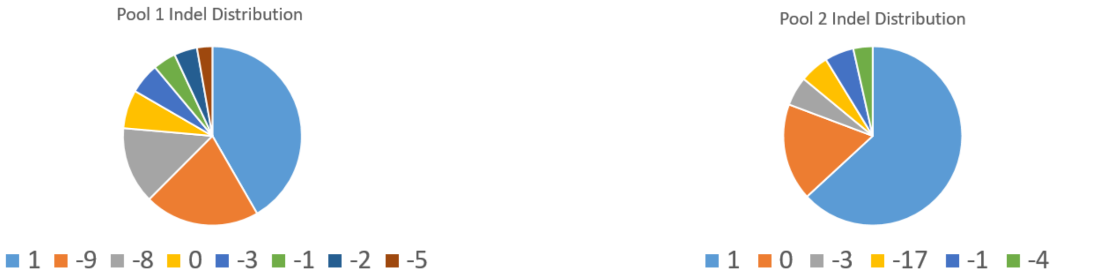

Figure S1.

A. Genomic DNA from edited pools was spiked-in to control wild-type DNA at increasing amounts and assayed using TIDE, NGS, IDAA, and T7E1. B. Indel distributions for top indels (2% or greater) is shown.

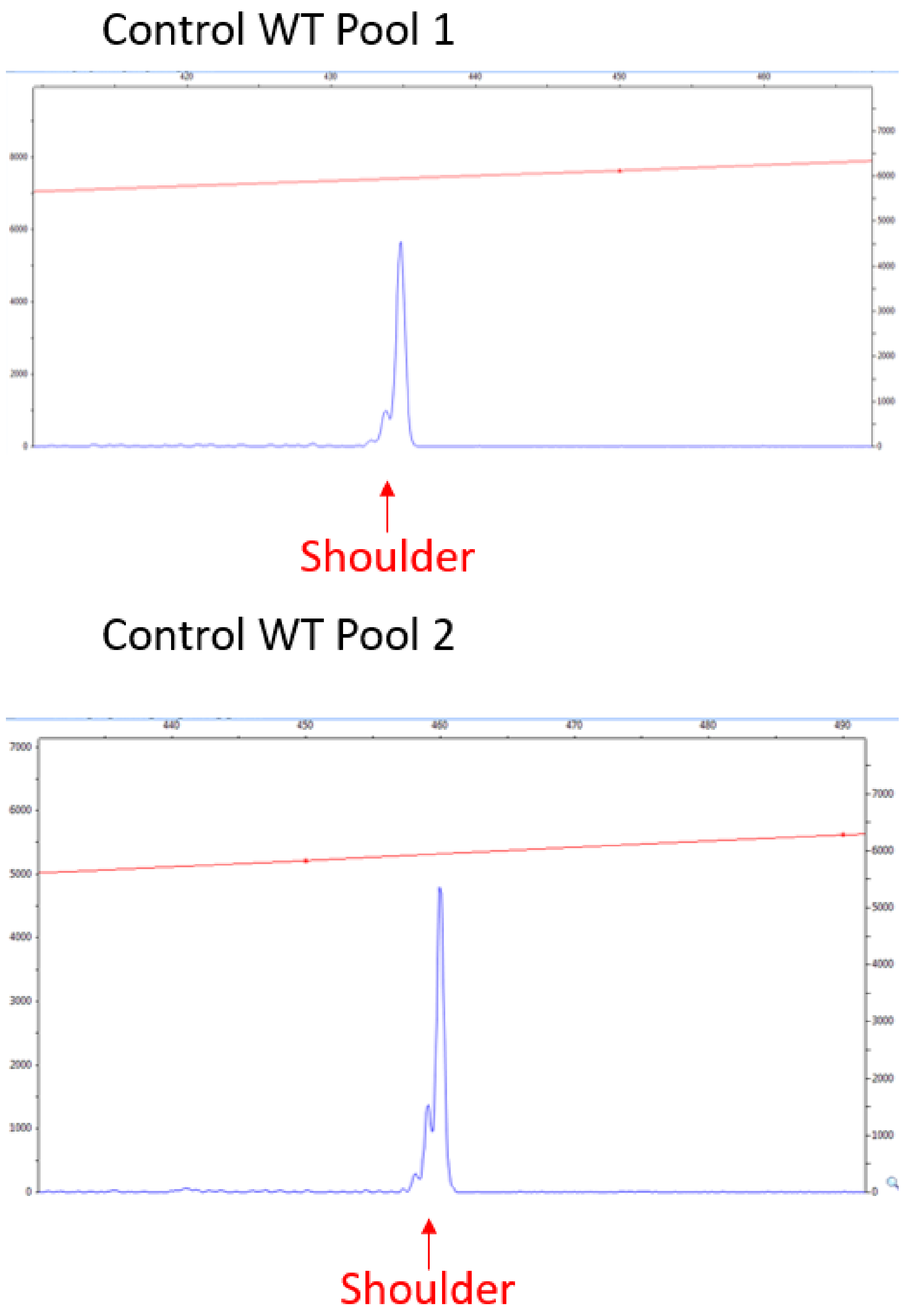

Figure S2.

IDAA example plots of pools 1 and 2 (Fig S1) highlighting shoulder.

Uncropped gels

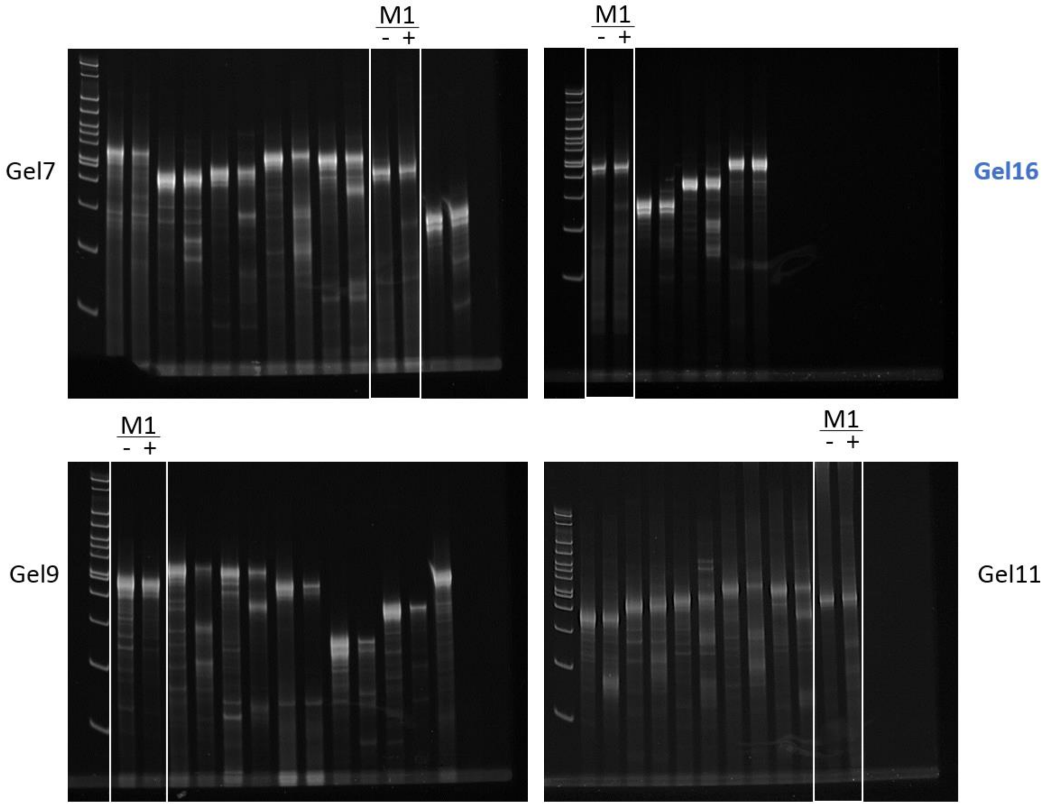

Fig1A

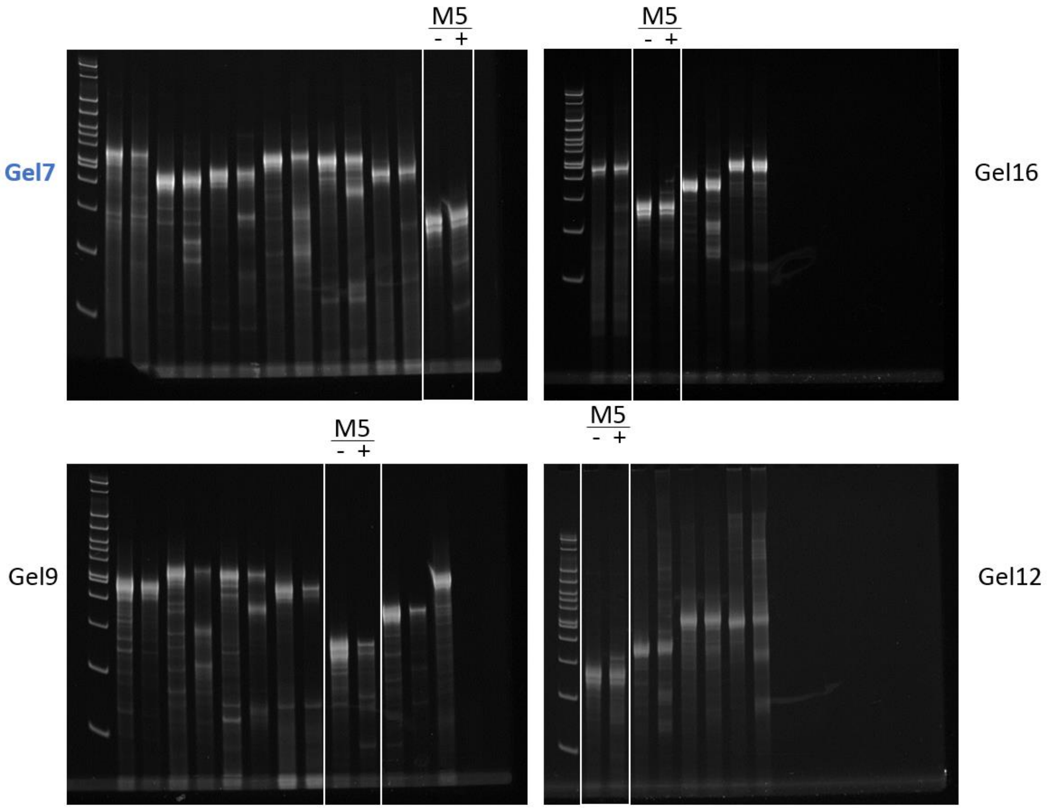

Fig1A

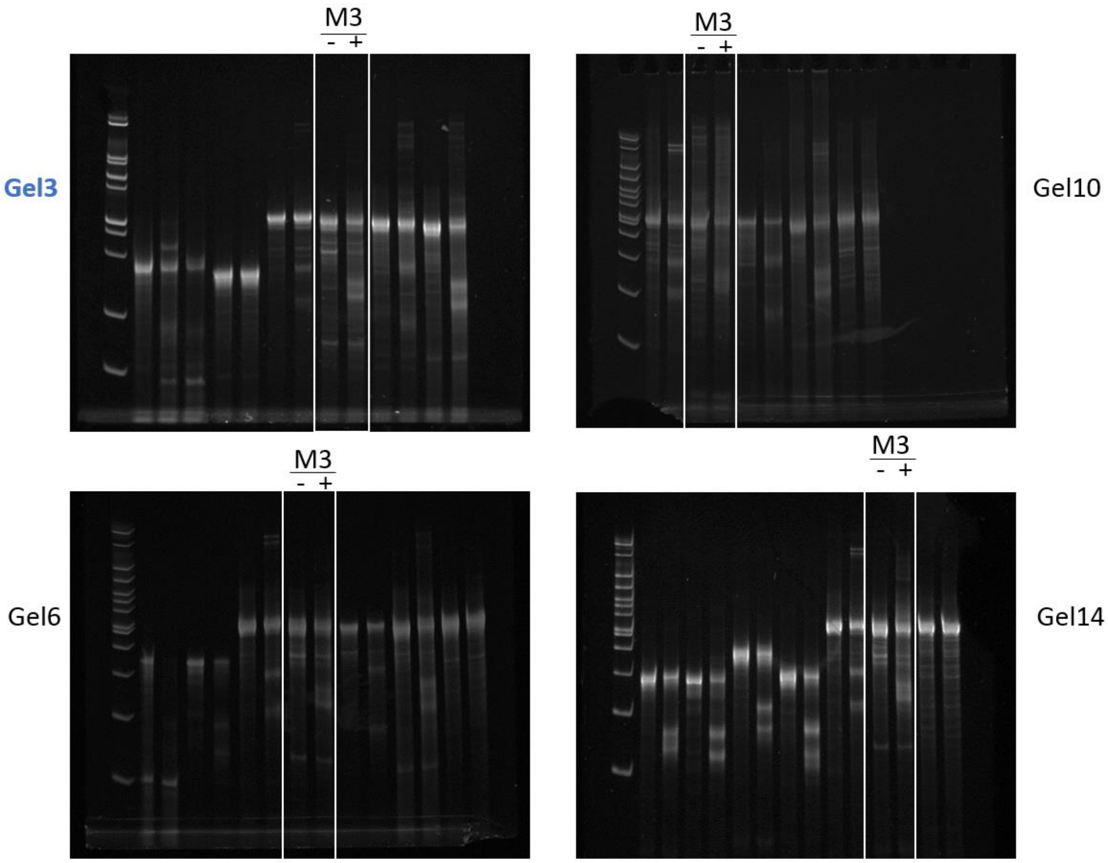

Fig1A

Gel2

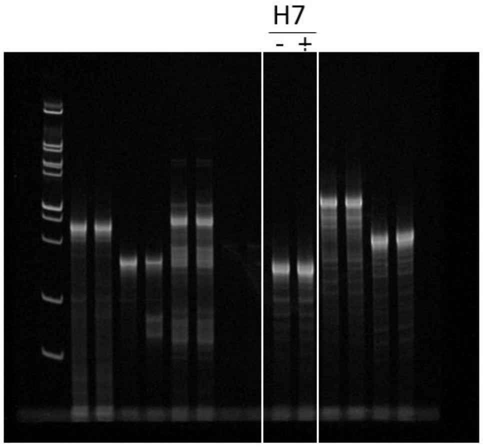

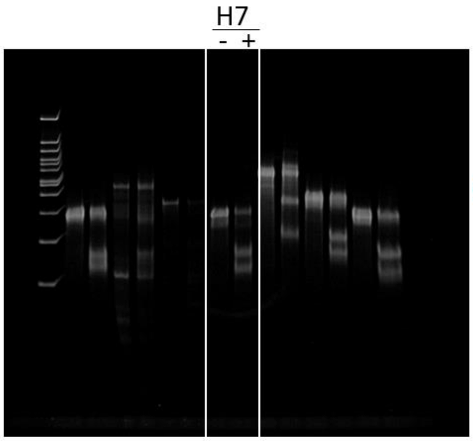

Gel19

Gel5

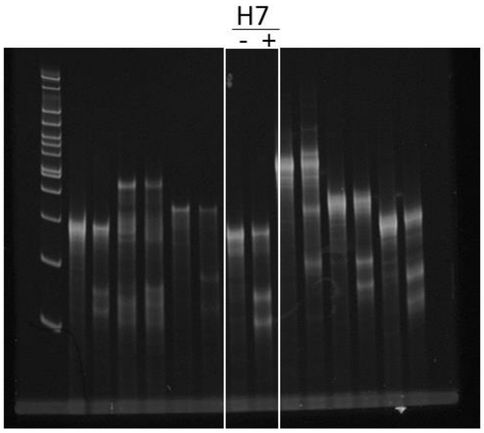

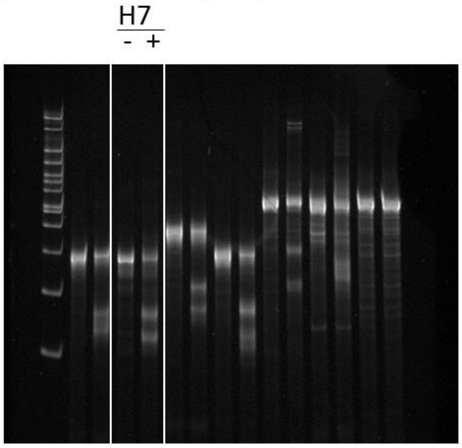

Gel14

Fig1A

Gel3

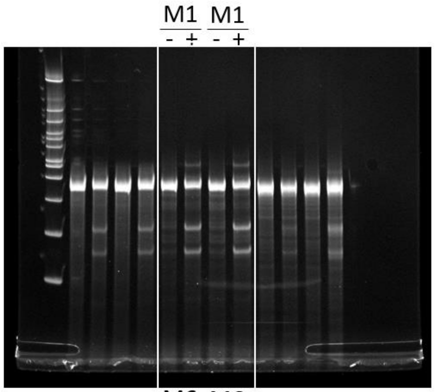

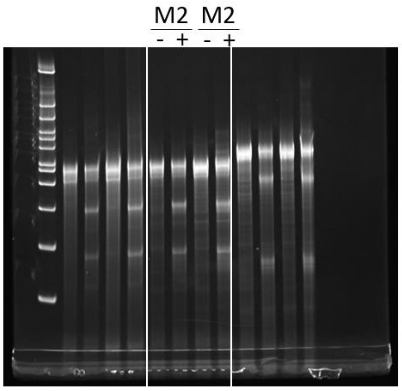

Gel2

Gel1

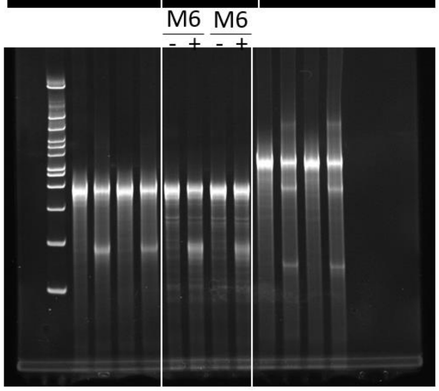

Exposure 5 sec – Higher contrast

Fig3B, 2 biological reps side-by-side

Gel3

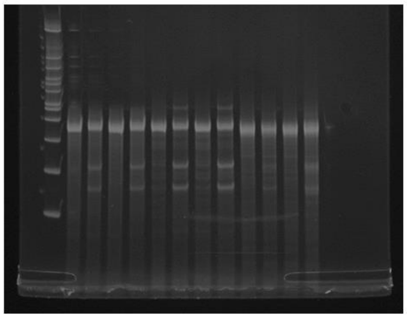

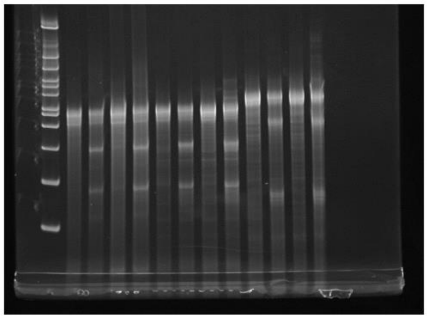

Gel2

Gel1

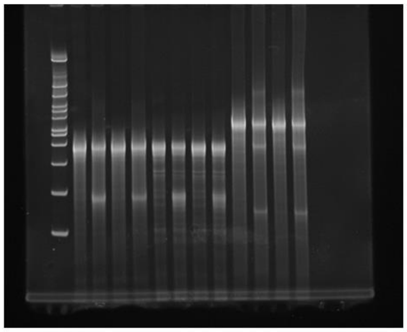

Exposure 5 sec - Regular

Fig3B, 2 biological reps side-by-side

Rep 1 Rep 2

Rep 3

Loci 1

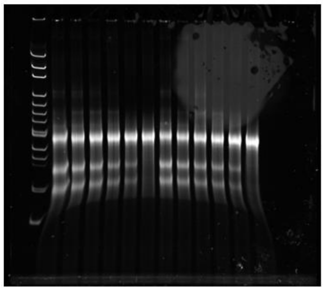

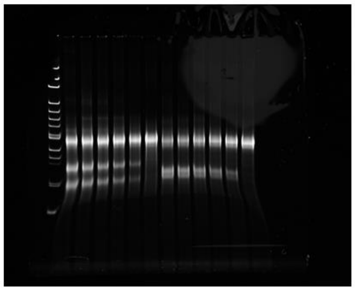

Rep 1 Rep 2

Rep 3

Loci 2

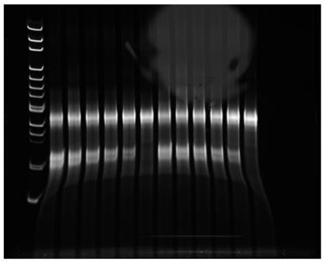

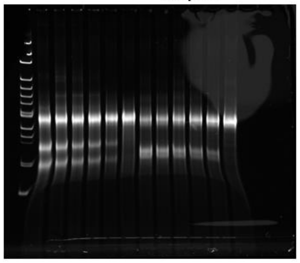

Exposure 5 sec – Supplemental Figure 1 (T7E1)
